# Antiadhesive Properties of Imidazolium Ionic Liquids Based on (−)-Menthol Against *Candida* spp.

**DOI:** 10.3390/ijms22147543

**Published:** 2021-07-14

**Authors:** Jakub Suchodolski, Joanna Feder-Kubis, Anna Krasowska

**Affiliations:** 1 Department of Biotransformation, Faculty of Biotechnology, University of Wrocław, 50-383 Wrocław, Poland; anna.krasowska@uwr.edu.pl; 2 Faculty of Chemistry, Wrocław University of Science and Technology, Wybrzeże Wyspiańskiego 27, 50-370 Wrocław, Poland; joanna.feder-kubis@pwr.edu.pl

**Keywords:** *Candida* spp., biofilm, ionic liquids, dentures

## Abstract

Infections with *Candida* spp. are commonly found in long-time denture wearers, and when under immunosuppression can lead to stomatitis. Imidazolium ionic liquids with an alkyl or alkyloxymethyl chain and a natural (1*R*,2*S*,5*R*)-(−)-menthol substituent possess high antifungal and antiadhesive properties towards *C. albicans*, *C. parapsilosis*, *C. glabrata* and *C. krusei*. We tested three compounds and found they disturbed fungal plasma membranes, with no significant hemolytic properties. In the smallest hemolytic concentrations, all compounds inhibited *C. albicans* biofilm formation on acrylic, and partially on porcelain and alloy dentures. Biofilm eradication may result from hyphae inhibition (for alkyl derivatives) or cell wall lysis and reduction of adhesins level (for alkyloxymethyl derivative). Thus, we propose the compounds presented herein as potential anti-fungal denture cleaners or denture fixatives, especially due to their low toxicity towards mammalian erythrocytes after short-term exposure.

## 1. Introduction

Fungi of the *Candida* spp. form microbial communities on human skin, and in the oral and genitourinary niches, with the posterior tongue and oral mucosa being the primary residence [1]. Within these niches, the commensal existence of *Candida* spp. varies by up to 75% in a healthy human population [2]. Under immunosuppression, *Candida* spp. are responsible for opportunistic infections, including life-threatening systemic fungaemias [3]. Chronic oral stomatitis associated with *Candida* spp. is common among patients with HIV, cancer or type 2 diabetes mellitus [4,5,6,7,8,9], although 50–70% of healthy denture wearers also suffer from *Candida*-associated denture stomatitis (CADS) [4,10,11]. CADS is predominantly caused by *C. albicans*; however *C. parapsilosis*, *C. glabrata*, *C. krusei*, *C. tropicalis* and *C. dubliniensis* have also been isolated from diseased tissues [12].

As the infection spreads, a biofilm grows progressively over the denture surface, leading to inflammation of the denture-exposed palatal mucosa and severe pain [11]. Fungal biofilms are encapsulated within a matrix of exopolymeric substance and have been shown to resist antimicrobials and immune host response [13,14]. Inhibition of *Candida* biofilm formation on denture materials is the first step towards the prevention of CADS [15].

The treatment of CADS focuses on sanitization of an existing, or fabrication of a new denture [16]. Oral antifungal agents such as amphotericin B, nystatin, miconazole, ketoconazole, itraconazole and chlorhexidine have been used for this purpose [13,15,17], but with limited to no effect [15]. Azoles are not effective in denture biofilm eradication [17,18] and once administered, the intraoral concentrations of antifungal drugs tend to be transient due to a diluent effect of saliva and cleansing effect of the oral muscles [19]. Prophylaxis with antifungal agents leads to the emergence of resistant strains [12,20], especially to azole antifungals [21].

New strategies that can either prevent fungal adhesion, or dislodge previously formed biofilms are increasingly needed [22]. Ionic liquids (ILs), such as those containing ammonium, pyridinium or imidazolium cations are effective as anti-microbial agents against bacterial rods, cocci and bacilli, and also fungi [23,24,25,26]. Recently, ILs based on the imidazolium cation have been patented and reported to act as anti-fungal mouthwashes, denture cleaners and denture fixatives. Furthermore, they can be used to prepare antimicrobial ion delivery systems to clean dentures and dentistry equipment, and can be added to dental tissue conditioner to improve its antifungal activity [27,28,29].

The presence of (1*R*,2*S*,5*R*)-(−)-menthol, a natural monoterpene alcohol, in the structure of ILs may significantly enhance their antimicrobial properties [30]. Previously, we have described strong antifungal and anti-adhesive properties of ammonium ILs based on (1*R*,2*S*,5*R*)-(−)-menthol towards *C. albicans* [31]. Taking into account the positive results of imidazolium ILs in dentistry [27,28,29], and our previous microbial tests highlighting strong antifungal and anti-adhesive properties of ammonium ILs with (−)-menthol moieties [31], we found it was justified to test ionic compounds containing a combination of the imidazolium cation with the monoterpene discussed in here.

Herein, we selected alkylimidazolium and alkoxymethylimidazolium chlorides, another group of (1*R*,2*S*,5*R*)-(−)-menthol-based ILs, in which the cationic region is based on the heterocyclic imidazole ring (all structures information are given in methodology section, Section 5.2). Our aim was to evaluate their antifungal and anti-adhesive properties on pathogenic *Candida* spp. and investigate their influence on *C. albicans* biofilm development on dentures. 

## 2. Results

### 2.1. Antifungal and Antiadhesive Properties of ILs

Table 1 summarizes the minimal inhibitory and fungicidal concentration (MIC_90_ and MFC, respectively) values of ILs tested against *Candida* spp. (for ILs structures see Section 5.2. No effect of the alkyl chain length (**1a** and **1b**) was observed to influence any inhibitory activity against all tested fungi. However, MFC values were two-fold lower for the compound with longer alkyl chain (**1b**) in each case. The compound with alkyloxymethyl substituent (**2a**) displayed reduced inhibitory activity when compared with salt structures containing alkyl residues. No differences were seen for MIC_90_ values of **1a** and **1b** against *C. albicans*, *C. parapsilosis* and *C. glabrata*, which seems justified as those compounds differ by only one CH_2_ group in the alkyl chain. Both **1a** and **1b** displayed an MIC_90_ two-fold lower against *C. krusei*. Salts containing a nonyloxymethyl substituent (**2a**) displayed an identical MIC_90_ value against all tested fungi. The MFC value of ILs with nonyl substituent (**1a**) is two- (*C. glabrata*) or four- (*C. albicans*; *C. parapsilosis*; *C. krusei*) fold higher than that of the MIC_90_ towards corresponding fungi. The MFC value of ILs with decyl substituent (**1b**) is identical (*C. parapsilosis*; *C. glabrata*; *C. krusei*) or two-fold higher (*C. albicans*) than that for the MIC_90_ towards corresponding fungi. Finally, the MFC value of **2a** is identical (*C. glabrata*) or two-fold (*C. albicans*; *C. parapsilosis*; *C. krusei*) higher than that of the MIC_90_ value (Table 1). The tested ILs were ranked as less active than a known antifungal drug, amphotericin B (AMB). No differences were seen for both MIC_90_ and MFC values (0.25 and 0.5 µM, respectively) of AMB against *C. albicans*, *C. parapsilosis* and *C. glabrata*. In case of *C. krusei*, both MIC_90_ and MFC values were two-fold higher than for the other *Candida* spp.

**Figure 1:**
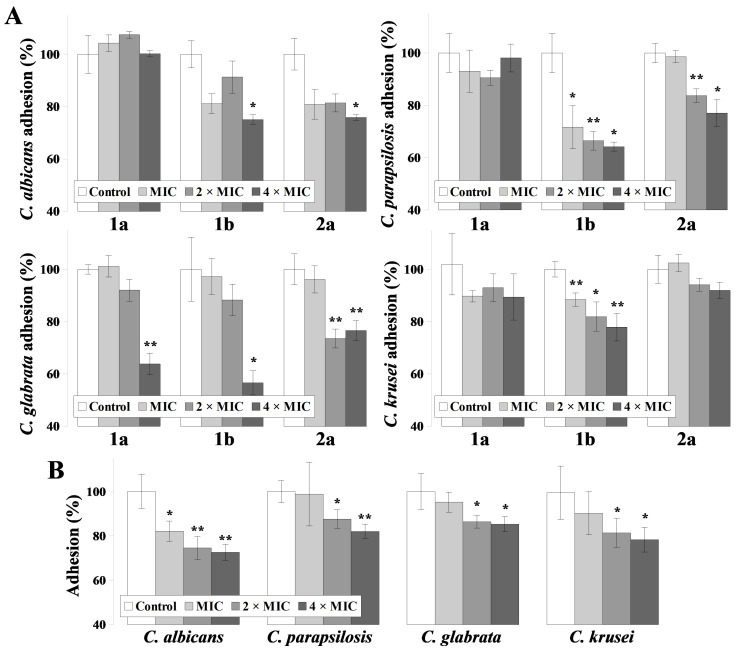
Detachment of adherent *Candida* spp. cells after 2 h incubation on polystyrene surfaces by ionic liquids (ILs) (**A**) or amphotericin B (AMB) (**B**) (means ± SD; *n* = 3). Results are presented as percentage of adherent cells relative to untreated controls (100% adhesion). Statistical analysis of detachment at each concentration was performed towards corresponding control experiments (fungi untreated by ILs or AMB = 100% adhesion) (*, *p* < 0.05 and **, *p* < 0.01).

Cells from adherent *Candida* spp. exhibit increased resistance towards antimycotics and the host immune system than planktonic cells. Thus, for the evaluation of ILs on fungal adhesion, higher concentrations were used, corresponding to 1, 2 and 4 times the MIC_90_ values (Figure 1).

IL with nonyl substituent (**1a**) did not influence the adherent properties of *C. albicans* or *C. parapsilosis,* but in the highest concentrations reduced *C. glabrata* adhesion by ~40% (*p* = 0.0012). Furthermore, *C. krusei* adhesion was reduced by approximately 10%, regardless of **1a** concentrations, although these changes were not significant (*p* = 0.21; 0.32; 0.22 for MIC, 2 × MIC and 4 × MIC, respectively). IL with alkoxymethyl chain (**2a**) reduced the adhesion of *C. albicans* in ~20–30% (*p* = 0.047 for 4 × MIC conc.); *C. parapsilosis* in ~30–40% (*p* = 0.012; 0.007; 0.011 for MIC, 2 x MIC and 4 × MIC, respectively); *C. glabrata* adhesion was reduced by approximately 45% when present at highest concentrations (*p* = 0.016), and *C. krusei* by approximately 10–20% (*p* = 0.007; 0.015; 0.007 for MIC, 2 × MIC and 4 × MIC, respectively). IL with decyl substituent (**1b**) did not significantly reduce *C. krusei* adhesion and displayed lower activity than **1a** and **2a** against *C. glabrata* ~20% reduction after treatment with 2 × MIC and 4 × MIC conc. *(p* = 0.005 and 0.007, respectively). Chloride salt **1b** displayed a higher than **1a** and lower than **2a** activity in *C. parapsilosis* adhesion reduction (~20–25%, *p* = 0.005 and 0.03 for 2 × MIC and 4 × MIC, respectively) and similarly to **2a** reduced *C. albicans* adhesion in ~20–25% (*p* = 0.04 for highest concentration). The activity of IL **2a** was comparable to the properties of AMB in case of *C. albicans* (~27% reduction in highest concentration, *p* = 0.002), *C. parapsilosis* (~20% reduction in highest concentration, *p* = 0.3) and *C. krusei* (~20% reduction in highest concentration, *p* = 0.03). However, IL **2a** displayed higher activity towards *C. glabrata* than AMB (~15% reduction in highest concentration, *p* = 0.03).

### 2.2. The Activity of ILs in Permeabilization of Fungal and Erythroid Membranes

We tested ILs for their activity in permeabilizing fungi plasma membranes (PMs), by treating cells with concentrations of ILs corresponding to their MIC_90_ values (Table 2). Chloride salt with nonyl substituent (**1a**) displayed high activity in PM permeabilization of *C. krusei* (*p* = 0.03), moderate activity for *C. albicans* (however, not statistically significant, *p* = 0.14) or *C. glabrata* (*p* = 9.9 × 10^−4^) and no activity towards *C. parapsilosis*. Chloride salt with nonyloxymethyl substituent (**2a**) displayed no activity (*C. parapsilosis*), moderate activity (*C. albicans*, *p* = 3.8 × 10^−4^; *C. krusei*, *p* = 0.016) and high activity (*C. glabrata*, *p* = 0.007) for PM permeabilization. No permeabilization was detected after treating *C. albicans* cells with chloride salt with decyl substituent (**1b**), statistically significant (*p* = 0.046) permeabilization was observed for *C. krusei* and moderate activity for *C. parapsilosis* and *C. glabrata* (*p* = 0.011 and 0.02, respectively). The tested ILs were ranked as less active in fungal PM permeabilization than AMB. The treatment with AMB resulted in ~40% permeabilization in case of *C. albicans* (*p* = 2.3 × 10^−4^), *C. glabrata* (*p =* 1.1 × 10^−4^) and *C. krusei* (*p* = 0.026), or ~30% in case of *C. parapsilosis* (*p* = 6.7 × 10^−5^).

To examine any possible toxic effect towards animal cells, ILs were tested for their lytic activity on sheep erythroid PMs, in comparison to AMB (Table 3). For this part of study, higher concentrations were selected, corresponding to MFC values of tested fungi (12.5; 25 and 50 µM in case of ILs, and 0.25; 0.5 and 1 µM in case of AMB, Table 1). The compounds were ranked in terms of hemolytic activity as follows: **1a** > **1b** > **2a**. IL with alkoxymethyl chain (**2a**) produced <5% hemolysis, regardless of the concentration used. Compounds with alkyl substituents (**1a** and **1b**) were more hemolytic in 12.5 than in 25 µM concentrations, and in the highest concentration (50 µM), both compounds produced >10% and >5% hemolysis, respectively. AMB, on the other hand, displayed high toxicity towards erythrocytes, resulting in almost 90% hemolysis in the lowest concentration, and total hemolysis in higher concentrations (Table 3).

### 2.3. ILs Prevent C. Albicans Biofilm Formation on Dentures

**Figure 2:**
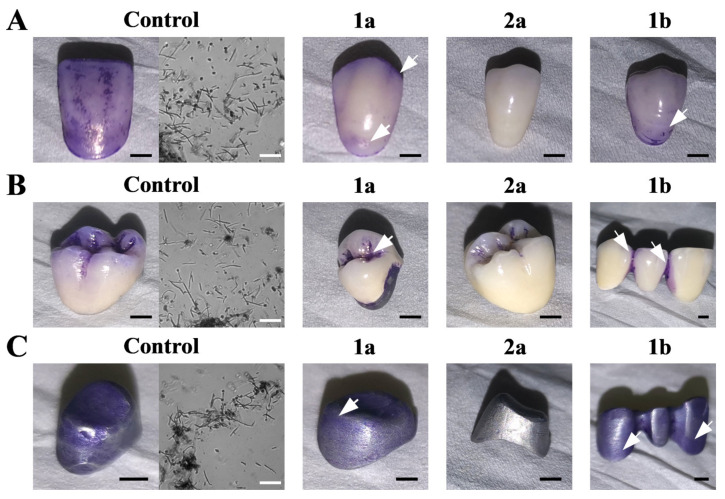
*C. albicans* biofilm formation visualized by crystal violet (CV) dye on. (**A**) acrylic dental crowns. (**B**) PFM dental crowns and (**C**) dental alloy substructures. Scale bar = 2 mm. Biofilm mass formed on control probes was observed under microscope (40×), scale bar = 50 µm. Samples were treated with ILs (**1a**,**2a**,**1b**) for 2 h to formed *C. albicans* biofilm. Arrows indicate partial biofilm formed on materials treated with ILs (**1a**) and (**1b**).

We evaluated the ability of ILs to eradicate *C. albicans* biofilm formation on a number of dentures, namely acrylic dental crowns, PFM dental crowns and dental alloy substructures (Figure 2). This approach has the following disadvantage: impossibility of reliable biofilm quantification. However, during in vivo *Candida* spp. infections and biofilm formation, the fungi attach themselves into porous structure of dentures, and thus we selected them for the studies over commonly used discs. Here, an IL concentration of 25 µM was selected for this investigation. In each condition, a *C. albicans* biofilm was formed after 72 h incubation, and the collected biomass was microscopically evaluated for the presence of hyphae, pseudohyphae and blastoconidia characteristic of *C. albicans* (Figure 2). Next, the materials were treated with ILs for 2 h and stained with crystal violet (CV). In the presence of salts with alkyl chains (**1a** and **1b**), residual CV staining was observed on each of the tested dentures (Figure 2). IL with alkyloxymethyl substituent (**2a**) protected acrylic dental crowns and dental alloy substructure from biofilm formation, but did not fully protect those made from PFM. Microscopic observations of the stained biomass from dentures exposed to both **1a** and **1b** (Figure 2, white arrows) confirmed the presence of *C. albicans* hyphae and blastoconidia (data not shown).

**Figure 3:**
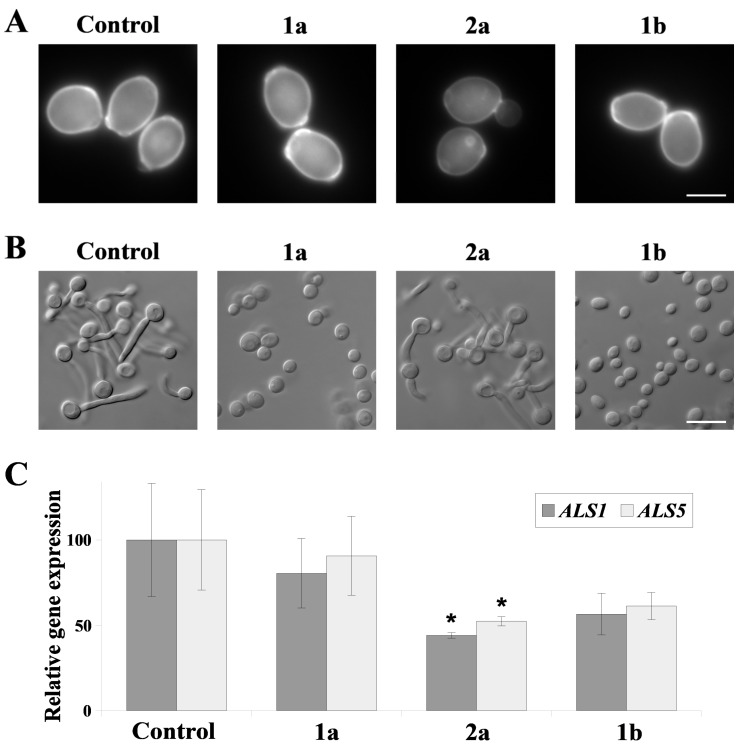
(**A**) *C. albicans* calcofluor white staining following a 2 h treatment with **1a**,**2a**,**1b** ILs (conc. = 25 µM). Control experiment represents untreated calcofluor white-stained cells. Scale bar = 2.5 µm. (**B**) FBS-induced hyphae formation after 2 h treatment with **1a**,**2a**,**1b** ILs (conc. = 25 µM). Control condition was *C. albicans* treated with FBS only. Scale bar = 10 µm. (**C**) Relative *ALS1* and *ALS5* gene expression in *C. albicans* following a 2 h treatment with **1a**,**2a**,**1b** ILs (conc. = 25 µM). Gene expression levels are reported as means of 2^−∆∆CT^ values (*n* = 6) ± SD; normalized to 100% for untreated cells (control). Statistical analyses were performed by comparing expression under treatment with each IL to untreated cells. * *p* < 0.05.

Fungal adhesion to biomedical surfaces is strongly related to the structure of the cell wall, and the presence of adhesins (Als proteins) on the surface of the cell. In turn, the progression to biofilm formation is dependent on the ability of *C. albicans* to form hyphae. In conditions similar to those above (ILs conc. = 25 µM), we examined *C. albicans* cell wall integrity, its ability to form hyphae, and the level of *ALS* genes expression, encoding Als1 and Als5 adhesins (Figure 3). Calcofluor white staining of *C. albicans* cells treated with **1a** and **1b** reveals well-defined, intense chitin fluoresce with clearly visible bud scars, comparable with the control staining (Figure 3A). After exposure to IL **2a**, *C. albicans* cells revealed low fluorescence intensity and thinner calcofluor white-stained chitin layer surrounding cells than the controls. In the presence of compounds with alkyl substituents (**1a** and **1b**), hyphae formation by *C. albicans* cells was completely inhibited (Figure 3B), and the percentage of cells forming hyphae was <1%. After treatment with IL **2a**, 78.2 ± 17.2% cells formed hyphae, significantly less when compared to the control conditions (94 ± 0.6% cell forming hyphae). Despite some reduction, the result was not statistically significant (*p* = 0.12). On the other hand, after the treatment with IL **2a**, the expression of *ALS1* and *ALS5* genes was reduced by 50 or 55%, respectively (Figure 3C). The results were statistically significant in both cases (*p =* 0.04 for either *ALS* gene). However, in case of compounds with alkyl substituents (**1a** and **1b**), the reduction of gene expression was to a lesser degree. The treatment with IL **1a** resulted in 10 or 20% reduction in case of *ALS5* or *ALS1* gene expression, respectively. It was observed that IL with longer alkyl substituent **1b** resulted in 40 or 45% reduction in case of *ALS5* or *ALS1* gene expression, respectively. Despite observing a chain-length dependence, the results were not statistically significant (*p* = 0.07 for both *ALS* gene after treatment with **1b**).

## 3. Discussion

In this research, three imidazolium-based ILs were chosen for investigations based on previous reports of strong antimicrobial properties of those compounds [23,24]. Moreover, imidazolium cation containing ILs are already used for denture cleaning applications [27,28,29]. The incorporation of natural terpene alcohol, (1*R*,2*S*,5*R*)-(−)-menthol in the structure of ILs follows the trend of researching more natural products in CADS treatment (such as: apple cider, probiotics, curcumin, tea tree oil or fulvic acid) [14,32,33]. 

No correlation was found between the alkyl chain length of the compounds and fungicidal activity for any of the tested fungi (Table 1); a similar observation was found in our previous studies on ammonium ILs based on (1*R*,2*S*,5*R*)-(−)-menthol towards the *C. albicans* SC5314 strain [31]. Here however, a positive correlation between the alkyl/alkoxymethyl chain length and fungicidal activity of the compounds was found (Table 1), which was not previously observed in the case of ammonium ILs [31]. Interestingly, imidazolium ILs are less active towards *C. albicans* than our previously tested ammonium ILs, with the alkyloxymethyl substituted compound being the least active in the current report (**2a**, Table 1). The cationic structures of the ILs salts with chloride anions and commonly used benzalkonium chloride (BAC) were previously ranked in terms of antimicrobial activity as follows: ammonium CILs > alkylimidazolium CILs > alkoxymethylimidazolium CILs > BAC > pyridinium CILs [30]. However, interspecies differences towards ILs were observed, especially with *C. glabrata* and *C. krusei* that were twice as vulnerable towards the fungicidal effects as *C. albicans* (MFCs, Table 2)*. C. glabrata* is known to be the second most prevalent *Candida* species, being isolated from acrylic denture surfaces and the palatal mucosa [34]. Since *C. glabrata* is more resistant to commercial denture cleaners and sodium hypochlorite (NaOCl) solutions than other *Candida* spp. [35], our result is especially promising.

ILs are generally considered cytotoxic, especially imidazolium derivatives [36], and thus should not be administered intravenously. However, here we evaluated the potential of the compounds as short-term eradicators of adherent cells or biofilm of *Candida* spp., in addition to their short-term safety towards mammalian erythrocytes, which are generally very vulnerable.

An essential step in the initiation of CADS and biofilm formation is the adhesion of *Candida* spp. to denture material [12]. Microbial adhesion on biomedical surfaces depends on the surface structure and composition of biomaterials, microbial cell surface hydrophobicity and specific interactions between cell surface structures and the surfaces of biomaterials [37,38]. For a tentative comparison of ILs activity in their ability to detach adherent *Candida* spp. cells, we examined their influence on cells adherent to polystyrene surface (Figure 1). Imidazolium (Figure 1) and ammonium [31] ILs reduced *C. albicans* adhesion in a comparable manner (20–30% reduction). Kanjanamekanant et al. [28] also demonstrated that IL-incorporated tissue conditioner reduces *C. albicans* adhesion to a similar degree (~25%). Despite the least fungistatic and fungicidal activities (Table 1), the alkyloxymethyl substituted compound (**2a**) was generally the most active against adherent cells (Figure 1). The results are especially promising since **2a** was the least hemolytic among the tested compounds (Table 3) and most (*C. albicans* and *C. glabrata*) or second most active (*C. krusei*) in PM permeabilization (Table 2).

The tested ILs displayed lower fungicidal, higher permeabilization and similar antiadhesive activities when compared to AMB—one of the “gold standards” during antifungal treatment (Figure 1, Table 1 and Table 2). The results represent in vitro situation, using common laboratory *Candida* spp. strains, and laboratory medium. However, during in vivo treatment of CADS, common antifungal agents such as AMB are not effective [15]. Additionally, AMB as a polyene drug binds to ergosterol in the fungal plasma membrane (PM), inducing permeabilization and cytoplasm leakage [39]. Polyenes also display specificity towards mammalian cholesterol, which results in high cytoxicity of those drugs, and the treatment is associated with a number of adverse effects among patients [39]. Here, this effect of AMB is represented by high hemolytic activity, which is not the case of the tested ILs (Table 3). Thus, despite lower in vitro activity of ILs towards *Candida* spp. than AMB, the usage of ILS in further studies seems justified—especially due to their activity towards clinical *C. albicans* isolates (Supplementary Materials). We have found that fluconazole (FLC)-resistant isolates are vulnerable towards the ILs in comparable manner as FLC-sensitive strains in either planktonic (Table S1) or adherent (Figure S1) form.

Similarly to previously examined ammonium ILs [31], a positive correlation was found between the alkyl chain length of the compounds (**1a** and **1b**) and antiadhesive activities towards *C. albicans* and *C. parapsilosis* (Figure 1). However, an opposite correlation was found in the hemolytic activity, as IL containing nonyl substituent (**1a**) was more hemolytic than the one with decyl substituent (**1b**) (Table 4). Previously we have demonstrated a similar feature of ammonium IL with decyl substituent and (−)-menthol moiety being more hemolytic than ammonium IL with dodecyl substituent [31]. The hemolytic properties of amphipathic molecules do not always correspond linearly to increasing alkyl chain length, as previously reported for quaternary ammonium salts [40]. The hemolytic activities depend not only on the hydrophobic moiety of the amphiphiles, but also on the size of the hydrophilic group, CMC and symmetry of the entire compound [41]. In yeast cells, intraspecies differences in PM composition affect resistance towards amphiphiles. As shown herein, ILs with nonyl substituent (**1a**) is more active in PM permeabilization of *C. albicans* and *C. krusei*, and **1b** is more active towards *C. parapsilosis* and *glabrata* (Table 2).

To be able to apply ILs in the protection of dentures from biofilm formation, only *C. albicans* strain was chosen, as this is the predominant cause of CADS and general microbial stomatitis [10]. In in vitro studies*, C. albicans* was shown to form a biofilm on a number of different denture biomaterials, including polymethyl methacrylate (PMMA, known as acrylic) and polyamide resins [13,15,35,42], resilient denture liners [17], polyvinyl chloride (PVC), polyurethane and teflon [43]. Here, we were able to observe the formation of *C. albicans’* biofilm on acrylic and PFM dental crowns, and dental alloy substructures (Figure 2). The concentration of ILs was chosen as 25 μM, since under such conditions ILs were the least hemolytic to mammalian erythrocytes (Table 3). All tested compounds visually eradicated biofilm that had formed on dentures (Figure 2). Similar results were observed for clinical, FLC-resistant isolates (Figure S2, Supplementary Materials). However, contrasting **1a** and **1b**, **2a** did not fully protect acrylic and alloy substructures, probably due to the highest antiadhesive properties for *C. albicans* (Figure 1). This is likely to occur through the ability to inhibit hyphae development (Figure 3). In vivo, *C. albicans* biofilms consists of networks of yeast cells and hyphae, embedded into cracks and imperfections of the dentures [13,14]. Similarly, we observed long hyphaes in biomass samples from the dentures (Figure 2, controls). Previously, we have identified high activities of alkyl substituted ammonium ILs in inhibiting *C. albicans* hyphae development [31]. However, alkyloxymethyl substituted ILs (**2a**) did not inhibit filamentation (Figure 2B), but displayed activity in cell wall lysis (Figure 2A). We hypothesize, that this ability of IL **2a** is also connected to reducing the presence of Als proteins on the cell surface of the fungi. Here, we observed that treatment of *C. albicans* cells with IL **2a** significantly reduced gene expression of two *ALS* genes (namely, *ALS1* and *ALS5*, Figure 3C). Both genes encode cell-surface glycoproteins, commonly known as adhesins, which are crucial for aggregative effects, and the attachment of the fungus to biotic and abiotic surfaces [44,45]. Thus, most likely due to this ability IL **2a** was the most effective in biofilm eradication from dentures (Figure 2).

Some strategies have been described to combat *Candida* biofilm in dentures, such as soaking in NaOCl, microwave irradiation, the application of effervescent cleansing tabs [18,46], preservatives and disinfectants [15] or treating surfaces with plasma [47]. However, harsh treatments may alter the surfaces of the biomaterials [15], and efficient treatment of CADS relies not only on the treatment of the infected tissues, but also on preserving the denture [48]. In our case, no alterations in denture structures were observed after treatment with ILs (Figure 2). 

In the human oral cavity, *Candida* spp. form mixed biofilms with streptococci and staphylococci bacteria [10,49]. The potential application of the presented compounds in situations resembling in vivo stomatitis (e.g., their activity against mixed *Candida*-bacteria biofilms) is promising, as all tested compounds were previously described to possess strong antibacterial properties [23,24]. However further development in this area is needed to examine their potential against biofilms of mixed *Candida* spp. and other bacterial species. 

## 4. Conclusions

The results from this study demonstrated high antifungal and anti-adhesive (especially **2a** and **1b**) properties of imidazolium-based ILs with (1*R*,2*S*,5*R*)-(−)-menthol towards pathogenic *Candida* spp. Additionally, presented ILs displayed low hemolytic activities, when compared to antifungal gold-standard, amphotericin B. For further studies, we were experimenting with *C. albicans* fungus. The tested ILs visually eradicated *C. albicans* biofilm formed on dentures made from different materials, partially in case of **1a** and **1b** and totally in case of compound **2a**. Biofilm eradication in case of **1a** and **1b** was correlated with inhibiting hyphae formation. Additionally, **1a** seem not to affect the adhesion phenotypically or *ALS* genes expression, whereas **1b** affects both processes. In case of **2a** the effect of eradicating *C. albicans* biofilm on dentures was connected to fungal cell wall lysis and reduction of adhesins (**2a**). Based on this research, it can be concluded that the compounds presented here may possess a potential as anti-fungal denture cleaners or denture fixatives (especially **2a**, a strong biofilm eradicator), due to their high antifungal activities, and low toxicity towards mammalian cells in short-term exposure.

## 5. Materials and Methods

### 5.1. Chemicals

Chemicals and reagents used in this study were purchased from the following sources: sodium dodecyl sulfate (SDS), crystal violet (CV), phosphate-buffered saline (PBS), and calcofluor white (CFW) (Sigma-Aldrich, Poznań, Poland); d-glucose, bacteriological agar, propidium iodide (PI), and fetal bovine serum (FBS) (manufacturer: Bioshop and distributor: Lab Empire, Rzeszów, Poland); yeast extract (YE) and peptone (manufacturer: BD and distributor: Diagmed, Warszawa, Poland). All chemicals were analytical grade purity.

### 5.2. Preparation of ILs

The syntheses of 3-alkyl-1-[(1*R*,2*S*,5*R*)-(−)-menthoxymethyl]imidazolium chlorides (**1a** and **1b**) abbreviated [C_n_-Im-C_1_OMen][Cl], where *n* = 9 or 10, were described in our previous publication [23]. 1-(1*R*,2*S*,5*R*)-(−)-Menthoxymethyl-3-nonyloxymethyl imidazolium chloride (**2a**), abbreviated as [C_1_OC_9_-Im-C_1_OMen][Cl] was published by us previously [24]. The synthesized quaternary imidazolium chlorides were characterized by nuclear magnetic resonance (^1^H NMR and ^13^C NMR). The results were comparable with those presented previously by our group [23,24]. Elemental analyses were performed for the obtained chlorides (**1a**,**1b**,**2a**), and the results are given in Table 4.

### 5.3. Strains and Growth Conditions

*C. albicans* SC5314 was a kind gift of Prof. D. Sanglard (Lausanne, Switzerland) [50]. *C. parapsilosis* CBS10947 was a kind gift of Dr K. Góralska (Łódź, Poland) while *C. glabrata* ATCC90030 and *C. krusei* ATCC6258 were kind gifts of Dr M. Dyląg (Wrocław, Poland). Clinical *C. albicans* isolates (description in Supplementary Materials) were kind gifts of Prof. S. Milewski (Gdańsk, Poland) and prof. J. Morschhäuser (Wurzburg, Germany). Fungal strains were routinely grown at 28 °C on YPD medium (2% glucose, 1% peptone, 1% YE) with agitation (120 rpm). Agar (2% final concentration) was used for solidification of the medium.

### 5.4. Determination of Minimal Inhibitory and Fungicidal Concentrations (MICs and MFCs)

Experiments were performed according to the Clinical and Laboratory Standards Institute (2008), 3rd ed. M27-A3 [51], with modifications described previously [31]. Briefly, MICs were determined by serially diluting ILs in YPD medium in sterile 96-well plates (Sarstedt) and inoculating with fungal suspensions (final OD_600_ per well = 0.01). After incubation (48 h; 28 °C), the OD_600_ was measured (ASYS UVM 340 Biogenet). The concentrations of ILs, which resulted in ≥90% growth inhibition, were determined as MICs. Each well was then replated on YPD agar and incubated for 5 days at 28 °C. The lowest concentrations of ILs, which resulted in a complete absence of fungal growth were determined as MFCs.

### 5.5. Antiadhesive Properties of ILs

Post-adherence treatment with ILs was performed in a similar manner to that described previously by our team [31,52]. *Candida* spp. suspensions were prepared by centrifuging YPD-grown cultures and resuspending fungal mass in PBS to an OD_600_ = 0.6. Sterile 96-well plates were inoculated with the prepared suspensions and incubated for 2 h at 37 °C to induce fungal adhesion. Afterwards, adherent cells were washed with PBS to remove planktonic cells and treated with ILs (concentrations equivalent to 1, 2 or 4 × MIC_90_ for each fungi) for 2 h at 37 °C. Then, each well was washed with PBS and adherent cells were fixed with 0.1% CV solution for 5 min, and washed with PBS. Cells were then treated with a solution of isopropanol 0.04 N HCL with 0.1% SDS to permeabilize adherent cells and release the CV dye. The absorbance of CV was measured at 590 nm (ASYS UVM 340 Biogenet). The percentage of cells that were removed was determined in comparison to adherent cells treated with PBS only (designated 100% adhesion).

### 5.6. Propidium Iodide (PI) Staining

The assessment of PM permeability was performed as described previously [53], with modifications. Briefly, 3 mL of *Candida* spp. cell suspensions (OD_600_ = 0.1) in PBS were mixed with ILs (concentrations equivalent to MIC_90_ against each fungi). Samples were incubated for 2 h at 37 °C, washed with PBS and stained with PI to the final dye concentration of 6 × 10^−6^ M for 5 min. Then, cell suspensions were washed with PBS and observed under the Zeiss Axio Imager A2 microscope equipped with the Zeiss Axiocam 503 mono microscope camera and Zeiss HBO100 mercury lamp. The percentage of permeabilized cells was evaluated by counting PI positive cells out of one hundred in three independent repetitions for each experiment. 

### 5.7. Hemolysis Assay

ILs were tested for hemolytic activity, as described previously [31]. Briefly, 5 mL of fresh sheep blood was centrifuged (2000 rpm, 15 min), washed with PBS and resuspended in PBS to achieve hematocrit level of 50%. Red blood cells (RBCs) were treated with ILs (concentrations = 12.5, 25 and 50 µM) for 2 h at 37 °C. Afterwards, RBCs were centrifuged (2000 rpm, 5 min) and the absorbance of the supernatant was measured at 540 nm (ASYS UVM 340 Biogenet). The percentage of hemolysis was determined in comparison to the hemoglobin absorbance released by a 1% SDS solution (100% hemolysis).

### 5.8. Biofilm Formation on Dentures

To assess the influence of ILs on biofilm formation by *C. albicans* strain SC5314 on dentures, the following materials were used: acrylic dental crowns, porcelain-fused-to-metal (PFMs) dental crowns and dental alloy substructures. All dentures were kind gifts from Prof. M. Łukaszewicz (Wrocław, Poland). Prior to the experiments, the dentures were sterilized by 1 h incubation in 70% pure ethanol (EtOH) solution, washed with sterile water to remove EtOH residues and dried under aseptic conditions. The experiments were performed by aseptically placing dentures in YPD, supplemented with FBS, and inoculating them with YPD-grown *C. albicans* suspension (0.01% Vol/Vol). After 72 h of incubation at 37 °C, the dentures were treated for 2 h with the addition of ILs (conc. = 25 µM), washed with PBS and fixed with 0.1% CV solution for 10 min, then washed with PBS to remove the stain residue. The dentures were then dried and photographed. Microscopic preparations were made of biofilm samples which were imaged using the Zeiss Axio Imager A2 microscope equipped with Zeiss Axiocam 503 mono microscope camera.

### 5.9. The Impact of ILs on Yeast-to-Hyphae Transition

Experiments were performed as described before [54], with modifications. YPD-grown *C. albicans* were pelleted, washed with fresh YPD medium and resuspended in YPD medium (OD_600_ = 0.4). The suspensions were treated with ILs (conc. = 25 µM) and FBS (final conc. = 10%) for 2 h at 37 °C. The samples were observed under the Zeiss Axio Imager A2 microscope equipped with Canon PowerShot G10 camera for assessment of cell morphology. For quantitative estimation, cells of each assay were counted in three independent repetitions determining the percentage of hyphae forming cells.

### 5.10. Calcofluor White Staining

Calcofluor white was used to assess cell wall integrity in *C. albicans*, following our protocol [55]. YPD-grown *C. albicans* cells were pelleted, washed with PBS and treated with ILs (conc. = 25 µM) for 2 h at 37 °C. Afterwards, the samples were washed with PBS, stained with calcofluor white (final conc. = 25 µM) for 5 min. Then, cell suspensions were washed with PBS, pelleted and observed under the Zeiss (Poznań, Poland) Axio Imager A2 microscope equipped with Zeiss Axiocam 503 mono microscope camera and the Zeiss HBO100 mercury lamp.

### 5.11. Real Time Quantitive Polymerase Chain Reaction

YPD-grown *C. albicans* cells were pelleted, washed with PBS and treated with ILs (conc. = 25 µM) for 2 h at 37 °C. Next, the cells were pelleted, and concentrated (PBS; OD_600_ = 20). RNA was isolated using the Total RNA Mini Kit (A&A Biotechnology, Gdańsk, Poland). Synthesis of cDNA and calculation of gene expression levels were performed as previously described [56]. The following gene-specific primers were used: ACT1_F (5′-TCC AGC TTT CTA CGT TTC CA-3′), ACT1_R (5′-GTC AAG TCT CTA CCA GCC AA-3′), ALS1_F (5′-TGT TGG TGT GAC TAC TTC CT-3′), ALS1_R (5′-TGT ACC ACT GTG TCA AT-3′), ALS5_F (5′-CTC CAC TAG TTA TGG GGA TGT-3′) and ALS5_R (5′-TGA GGG AGA AAT ATA AGC GTC A-3′).

### 5.12. Statistical Analysis

Unless stated otherwise, data represent the mean ± standard errors from at least three biological replicates. Microscopic observations were performed for at least two independent replicates, of which the representative images were included in the figures. When indicated, statistical significance was determined by a Student’s *t*-test (binomial, unpaired).

## Figures and Tables

**Table 1:** Minimal inhibitory concentrations (MIC_90_; µM) and minimal fungicidal concentrations (MFC; µM) of ionic liquids (ILs) or amphotericin B (AMB) towards *C. albicans* SC5314, *C. parapsilosis* CBS10947, *C. glabrata* ATCC90030 and *C. krusei* ATCC6258.

Compound		*C. albicans*	*C. parapsilosis*	*C. glabrata*	*C. krusei*
**1a**	MIC	12.5	12.5	12.5	6.25
	MFC	50	50	25	25
**1b**	MIC	25	25	25	25
	MFC	50	25	25	25
**2a**	MIC	12.5	12.5	12.5	6.25
	MFC	25	25	12.5	12.5
**AMB**	MIC	0.25	0.25	0.25	0.5
	MFC	0.5	0.5	0.5	1

**Table 2:** Percentage of cells with permeabilized PMs after 2 h treatment with ILs or AMB (mean ± SD; *n* = 3). Statistical analysis was performed by comparing experiments of non-treated fungal cells (control conditions) with those treated with ILs or AMB. * *p* < 0.05; ** *p* < 0.01 and *** *p* < 0.001.

Condition/Compound	*C. albicans*	*C. parapsilosis*	*C. glabrata*	*C. krusei*
**Control**	3.12 ± 1.7	2.02 ± 0.72	1.43 ± 1.1	0.99 ± 0.67
**1a**	12.42 ± 6.88	2.74 ± 0.92	12.34 ± 0.46 ***	36.28 ± 11.54 *
**1b**	22.48 ± 2.19 ***	3.86 ± 0.99	42.15 ± 6.12 **	16.45 ± 7.84 *
**2a**	4.42 ± 0.92	21.29 ± 8.47 *	23.8 ± 5.92 *	9.6 ± 3.49 *
**AMB**	37.3 ± 4.17 ***	32.38 ± 3.15 ***	43.2 ± 2.15 ***	38.2 ± 10.7 *

**Table 3:** Hemolytic activity of ILs or AMB (%). The positive control of 1% SDS was designated as 100% hemolysis (mean ± SD; *n* = 3). Statistical analysis was performed by comparing experiments performed by spontaneous hemolysis in PBS to IL- or AMB-treated erythrocytes. * *p* < 0.05; ** *p* < 0.01 and *** *p* < 0.001.

	**ILs Conc. (µM)**			
**Compound**	**0 (Control)**	**12.5**	**25**	**50**
**1a**	1.09 ± 0.01	4.81 ± 0.02 **	1.51 ± 0.53	11.56 ± 2.13
**1b**	1.11 ± 0.02	2.1 ± 0.36	3.01 ± 0.39	3.23 ± 0.6
**2a**	1.07 ± 0.01	4.55 ± 0.82	1.78 ± 0.44	6.09 ± 0.48 *
	**AMB Conc. (µM)**			
	**0 (Control)**	**0.25**	**0.5**	**1**
**AMB**	1.34 ± 0.04	86.7 ± 0.43 ***	98.6 ± 1.53 ***	100 ± 0.73 ***

**Table 4:** Structure, name, abbreviation, empirical formula, yield, elementary analysis, both calculated and observed for tested ionic liquids with a natural (1*R*,2*S*,5*R*)-(−)-menthol moiety (**1a**,**1b**,**2a**).

	Structure, Name, Abbreviation, Empirical Formula	Yield [%]	Elementary Analysis [%]	
			Calculation	Found
**1a**	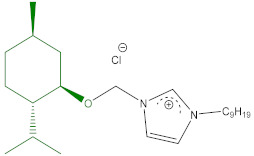 1-[(1*R*,2*S*,5*R*)-(−)-menthoxymethyl]-3-nonylimidazolium chloride [C_9_-Im-C_1_OMen][Cl] C_23_H_43_ClN_2_O	98.0 ^a^	C 69.22 H 10.86 N 7.02	C 69.31 H 10.92 N 6.90
**1b**	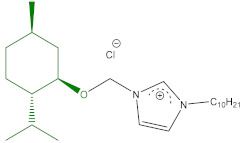 3-decyl-1-[(1*R*,2*S*,5*R*)-(−)-menthoxymethyl]imidazolium chloride [C_10_-Im-C_1_OMen][Cl] C_24_H_45_ClN_2_O	97.5 ^b^	C 69.78 H 10.98 N 6.78	C 69.69 H 11.08 N 6.82
**2a**	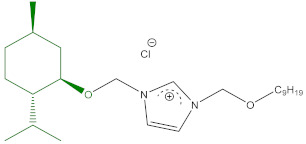 1-(1*R*,2*S*,5*R*)-(−)-menthoxymethyl-3-nonyloxymethylimidazolium chloride [C_1_OC_9_-Im-C_1_OMen][Cl] C_24_H_45_ClN_2_O_2_	96.0 ^c^	C 67.18 H 10.57 N 6.53	C 67.24 H 10.66 N 6.41

^a^ literature data yield for **1a** = 97% [23]; ^b^ literature data yield for **1b** = 97% [23]; ^c^ literature data yield for **2a** = 94.5% [24].

## Data Availability

The data presented in this study are available on request from the corresponding author (J.S.).

## Supplementary Materials

The following are available online at https://www.mdpi.com/article/10.3390/ijms22147543/s1, Table S1: Minimal inhibitory concentrations (MIC_90_; µM) and minimal fungicidal concentrations (MFC; µM) of ionic liquids (ILs) or fluconazole (FLC) towards clinical *C. albicans* isolates.; Figure S1: Detachment of adherent *C. albicans* clinical isolates after 2 h incubation on polystyrene surfaces by ionic liquids (ILs) (means ± SD; *n* = 3). Results are presented as percentage of adherent cells relative to untreated controls (100% adhesion). Statistical analysis of detachment at each concentration was performed towards corresponding control experiments (isolates untreated by ILs = 100% adhesion) (* *p* < 0.05; ** *p* < 0.01 and *** *p* < 0.001).; Figure S2: *C. albicans* clinical isolates (B4 and Gu5) biofilm formation visualized by crystal violet (CV) dye on acrylic dental crowns. Scale bar = 2 mm. Biofilm mass formed on control probes was observed under microscope (40x), scale bar = 50 µm. Samples were treated with ILs (**1a**; **2a**; **1b**) for 2 h to formed *C. albicans* biofilm.
