# Supplementary material for: Antiadhesive Properties of Imidazolium Ionic Liquids Based on (−)-Menthol Against Candida spp
Source: Int J Mol Sci. 2021 Jul 14;22(14):7543. doi: 10.3390/ijms22147543 (PMC8304783; doi:10.3390/ijms22147543)
Supplement: Supplementary file 1 [file ijms-22-07543-s001.zip › IJMS_ILs_Supplementary.pdf]

International Journal of
Molecular Sciences

MDPI

Supplementary materials

The effect of imidazolium ILs based on of (1*R*,2*S*,5*R*)-(−)-menthol was evaluated on clinical, fluconazole(FLC)-resistant and FLC-sensitive *C. albicans* isolates. *C. albicans* Gu4 and Gu5 strains were isolated from a patient before and after fluconazole administration, respectively. Azole-resistance of Gu5 origins in overexpression of *CDR1* and *CDR2*. *C. albicans* B3 and B4 strains were isolated from a patient before and after fluconazole administration, respectively. Azole-resistance of B4 results from overexpression of *MDR1*. The strains were generous gifts from Prof. S. Milewski (Gdańsk, Poland) and Prof. J. Morschhäuser (Wurzburg, Germany). They are originally referenced in: *Franz, R., Ruhnke, M. & Morschhäuser, J. Molecular aspects of fluconazole resistance development in Candida albicans. Mycoses 42, 453–458, (1999).*

Table S1 summarizes the minimal inhibitory and fungicidal concentration (MIC_{90} and MFC, respectively) values of ILs tested against the *C. albicans* isolates (for ILs structures see section 5.2. in the main text). No difference in case of MIC_{90} values between the strains was observed. However, MFC values were two-fold lower for the compounds **1b** and **2a** in case of B3 and B4 isolates. The MFC value of the compound **1a** was two-fold higher only in case of Gu5 strain.

Table S1. Minimal inhibitory concentrations (MIC_{90}; μM) and minimal fungicidal concentrations (MFC; μM) of ionic liquids (ILs) or fluconazole (FLC) towards clinical *C. albicans* isolates.

			***C. albicans* clinical isolate**		
		**Gu4**	**Gu5**	**B3**	**B4**
**1a**	MIC	12.5	12.5	12.5	12.5
	MFC	25	50	25	25
**1b**	MIC	25	25	25	25
	MFC	50	50	25	25
**2a**	MIC	12.5	12.5	12.5	12.5
	MFC	25	25	12.5	12.5

For the evaluation of ILs on the *C. albicans* isolates adhesion concentrations corresponding to 1, 2 and 4 times the MIC_{90} values were used (Figure S1). In most cases, IL **1a** did not influence the adherent properties of *C. albicans* isolates. Only in the highest concentrations **1b** have reduced Gu5, B3 and B4 adhesion by ~10% (not statistically significant). IL **1b** reduced the adhesion of Gu4 by ~20%, regardless of the concentration. Additionally, in higher concentrations (2 x MIC and 4 x MIC), **1b** reduced Gu5 and B4 adhesion by 15-20%. IL with alkoxymethyl chain (**2a**) reduced the adhesion of Gu4 and Gu5 ~20-35%, regardless of the concentration. In case of B3 and B4 strains, **2a** was active only in the highest concentration (4 x MIC), resulting in ~25% adherence reduction.

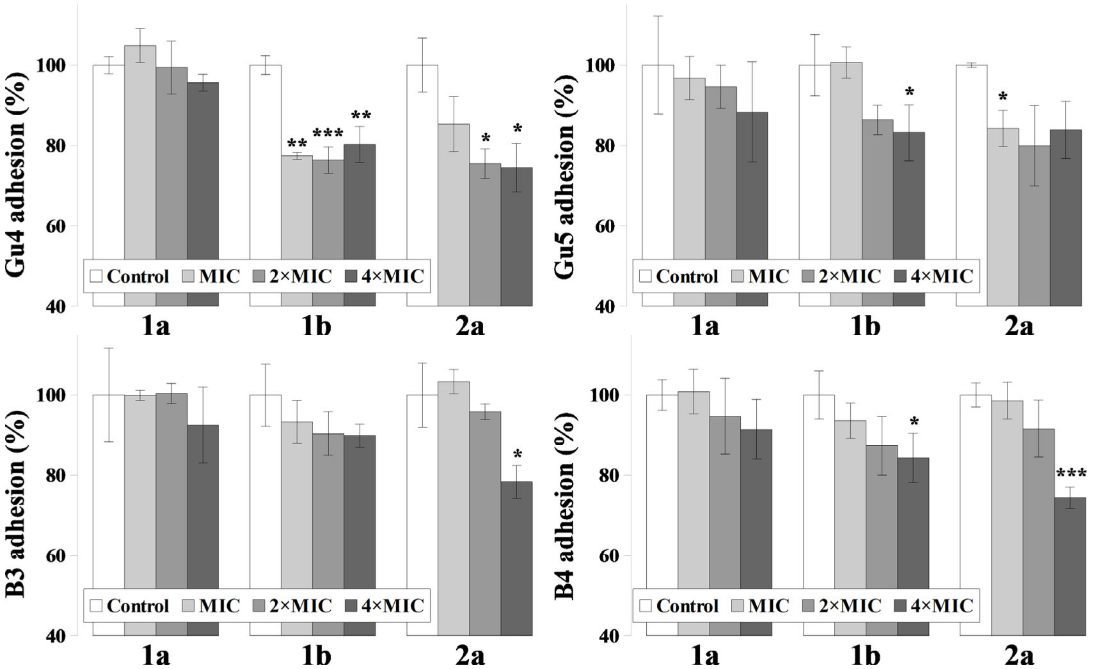

Figure S1. Detachment of adherent *C. albicans* clinical isolates after 2 h incubation on polystyrene surfaces by ionic liquids (ILs) (means ± SD; $n = 3$). Results are presented as percentage of adherent cells relative to untreated controls (100% adhesion). Statistical analysis of detachment at each concentration was performed towards corresponding control experiments (isolates untreated by ILs=100% adhesion) (*, $p < 0.05$; **, $p < 0.01$ and ***, $p < 0.001$).

We selected *C. albicans* B4 and Gu5 isolates for evaluating the ability of ILs to eradicate biofilm formation on acrylic dental crowns (Figure S2). Both the strains were chosen due to their FLC-resistance status. An IL concentration of 25 μM was selected for this investigation. In each condition, a *C. albicans* biofilm was formed after 72 h incubation, and the collected biomass was microscopically evaluated for the presence of hyphae, pseudohyphae and blastoconidia characteristic of *C. albicans* (Figure S2). Next, the materials were treated with ILs for 2 h and stained with crystal violet (CV). In the presence of salts with alkyl chains (**1a** and **1b**), residual CV staining was observed on each of the tested dentures. IL with alkyloxymethyl substituent (**2a**) protected acrylic dental crowns from biofilm formation.

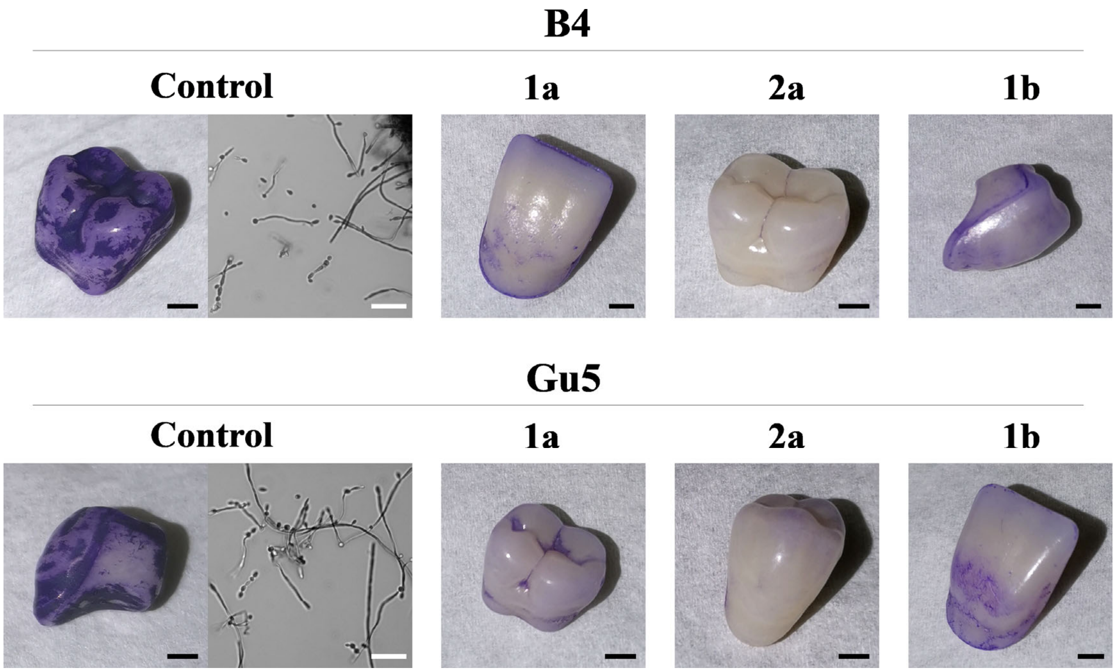

Figure S2. *C. albicans* clinical isolates (B4 and Gu5) biofilm formation visualized by crystal violet (CV) dye on acrylic dental crowns. Scale bar = 2 mm. Biofilm mass formed on control probes was observed under microscope (40x), scale bar = 50 μm. Samples were treated with ILs (**1a**; **2a**; **1b**) for 2 h to formed *C. albicans* biofilm.
